# 

**Published:** 1894-09

**Authors:** 


THE *Richmond Journal of Practice* is the new name evolved from the well-known *Practice* edited and published by Dr. John F. Winn, of Richmond, Va. It is a handsomely printed magazine of forty pages and contains the announcement of an able corps of collaborators. We wish the editor and his beautiful journal the abundant prosperity that he and it deserves.

---

DR. IRVING A. WATSON, editor of *Physicians and Surgeons of America*, has issued the following circular:

CONCORD, N. H., Aug. 12, 1894.

For the information of subscribers and others interested in *Physicians and Surgeons of America*, I have the pleasure to state that the work is progressing in a most satisfactory manner and as rapidly as is consistent with that scrupulous supervision necessary to insure accuracy.

I hope to have it ready for delivery in from six to eight months from this date. It will constitute the largest and most elegant volume on American medical biography yet issued, and will be illustrated by over a thousand first-class, half-tone copperplate portraits.

Patrons who have not already received a typewritten copy of their biography for correction will have the opportunity before their sketch goes to press.

---

THE F. A. Davis Company, of Philadelphia, will issue, in September, a work which undoubtedly will be most favorably received by the medical profession. It is entitled *Obstetric Surgery*, and is written by Drs. Egbert H. Grandin and George W.

Jarman, gentlemen who, from their long connection with the largest and most widely known maternity hospital in the United States (The New York Maternity Hospital), are peculiarly fitted to expound the subject from the modern progressive standpoint of election.

The work is not burdened with literature references. The authors have aimed to teach that which ample and prolonged experience has taught them is good. The net price of the volume will be $2.50, and it will be printed in large, clear type, on excellent paper, and handsomely bound in extra cloth. The full-page plates, about fourteen in number, will be printed on fine plate paper, in photogravure ink.

A companion volume, dealing in the same terse, practical manner with pregnancy, normal labor, and the physiological and pathological puerperium, is in active preparation by the same authors.

---

A Reading Idea for Invalids.—To make an envelope library, take ten envelopes, and put either a short story, an essay or illustrated article in each, writes Rose Crosby in an article describing an envelope library in the September *Ladies' Home Journal.* Lay the envelopes lengthwise before you, and rule off a space at the top in which to write the words, "Envelope Library No. I.," "Envelope Library No. II.," and so on through the series of ten. Rule off a space at the bottom in which to write the name of the story or article, and the author's name.

When the envelopes are filled, tie the ten together with a dainty ribbon, and send them where they will do the most good.

For use in hospitals these dainty packages of stories have proved very satisfactory. Weary convalescents, and especially those never visited by friends, are not only pleased with the gift, but are relieved from the fatigue that accompanies the holding of a heavy book or magazine.

---

In spite of the new medical journals that appear from time to time, the number of them remains about the same, for many drop out of the race and cease to live. Whether this is the survival of the fittest is not always certain. It is, however, certain that the present hard times are felt by many medical journals and the fact that so many continue to live and even that new ones are born in these troublesome times, shows great faith in expected support and reliance on the profession for help.—*Maryland Med. Journal.*

